# Effect of a fluoride-releasing fissure sealant and a conventional fissure sealant on inhibition of primary carious lesions with or without exposure to fluoride-containing toothpaste

**DOI:** 10.15171/joddd.2019.023

**Published:** 2019-08-14

**Authors:** Zahra Khalili sadrabad, Ebrahim Safari, Maryam Alavi, Mohammad Mostafa Shadkar, Seyed Hamid Hosseini Naghavi

**Affiliations:** ^1^Department of Pediatric Dentistry, Faculty of Dentistry, Tabriz University of Medical Sciences, Tabriz, Iran; ^2^Faculty of Physics, University of Tabriz, Tabriz, Iran; ^3^Private Practice, Tabriz, Iran; ^4^Private Practice, Tehran, Iran; ^5^Department of Periodontics, Faculty of Dentistry, Isfahan (Khorasgan Branch), Islamic Azad University, Isfahan, Iran

**Keywords:** Dental caries, fissure sealant, fluoride, toothpaste

## Abstract

***Background.*** Fluoride-releasing capacity has been added to fissure sealants to benefit from the positive anticariogenic effects of both sealants and fluoride. This comparative research investigated the inhibitory effects of conventional and fluoridereleasing fissure sealants on initial lesions with or without exposure to fluoride toothpaste.

***Methods.*** Cavities were prepared on buccal surfaces of 24 premolar teeth which were randomly divided into three groups. In the cavities of the first group, a fluoride-releasing fissure sealant and in the second group, a conventional fissure sealant were placed; the third group was left intact. Incipient lesions were produced around the cavities. Each group was divided into two subgroups, which were exposed to fluoride-containing toothpaste or artificial saliva. Lesion depths were measured under a polarized light microscope before and after treatment. Changes in lesion depths in the samples were analyzed by SPSS 17.

***Results.*** Initial and final caries depths were significantly lower in the fluoride-releasing fissure sealant group compared to the other groups (P<0.001). The average depths of carious lesions were lower in subgroups exposed to fluoride-containing toothpaste than the subgroups exposed to artificial saliva and the difference was significant in the conventional sealant group and the group without sealant (P<0.001); however, the difference between the toothpaste-exposed and saliva-exposed subgroups was not significant in the fluoride-releasing fissure sealant group (P=0.721).

***Conclusion.*** Incorporation of fluoride into the fissure sealants can be effective in the inhibition of dental caries. It seems that fluoride, released from fluoride-releasing sealants, overwhelms the remineralizing capacity of fluoride released from the toothpaste on the same tooth.

## Introduction

Dental caries is one of the most prevalent chronic diseases among children. Risk factors of dental caries include low fluoride levels in drinking water and foodstuff, living in a low-income family, and poor hygiene.^1^ If dental caries is not treated, it can lead to pain, infection and problems such as disorders in eating, learning and speaking.^2^

During the process of primary decay formation, demineralization of tooth enamel occurs. In general, an increase in remineralization and a decrease in demineralization of enamel depend on the fluoride, phosphate, hydroxide and calcium existing in saliva, and fluoride, especially its topical form, is an effective material in increasing remineralization of tooth enamel.^3^ Existence of fluoride ions with an adequate amount of calcium and phosphate ions in saliva can thermodynamically cause improved growth of crystals of tooth enamel and remineralization of demineralized enamel. Also, through disrupting metabolism of microorganisms existing in the dental plaque, fluoride ions inhibit acid production by microorganisms existing in the dental plaque.^3^ Continuous contact with low concentrations of fluoride leads to decreased demineralization of tooth enamel and an improvement in the process of remineralization.^4,5^ The effect of brushing teeth on preventing dental caries has been shown and there is convincing evidence on the anticariogenic effect of using fluoride toothpastes.^6^ Systematic reviews have shown that standard use of fluoride toothpaste reduces the rate of decay in permanent teeth by 24‒29%.^7^ In a study by Walsh et al,^8^ the significant effect of fluoride toothpaste with fluoride concentration of >1000 ppm on preventing primary dental caries was observed. In another study by Nazzal et al,^9^ the amount of fluoride in saliva had a significant relationship with an increase in the amount of fluoride in the toothpaste used (P˂0.001). In addition, it was concluded that use of toothpastes with fluoride concentrations of >1000 ppm for children leads to a decrease in the rate of incipient caries.

It has been shown that due to increased accumulation of plaque in pits and fissures of occlusal surfaces, dental caries occurs more easily on these areas.^10^ Pits and fissure sealants are useful and efficient in preventing dental caries on occlusal surfaces of molar and premolar teeth and therefore in preserving oral health.^11^ Fissure sealants are placed on areas that are susceptible to decay and with micromechanical bond, they act as a protective layer and prevent the access of bacteria to nutritious resources.^11^ Recently, fluoride releasing capacity has been added to fissure sealants in order to benefit from the positive effects of both the sealant and fluoride. It seems that incorporation of fluoride to pits and fissure sealants can be effective in decreasing caries of pits and fissures and in general, it leads to decreased prevalence of dental caries.^11,12^ After removal from the tooth surface, these materials continue to apply their anticariogenic effect microscopically. This effect can be caused by increased concentrations of fluoride on tooth enamel and also by these materials remaining in the depth of fissures.^13^ However, studies on the effects of fluoride-releasing fissure sealants on inhibition of demineralization have yielded contradictory results. A recent study demonstrated significantly higher inhibition of caries by fluoride-releasing sealants compared to conventional sealants.^14^

Prabhakar^11^ showed no significant inhibition of demineralization between fluoridated and non-fluoridated resin sealants. Moreover, no study has been carried out to compare the effects of fluoride-releasing and conventional fissure sealants in conjunction with exposure to fluoride-containing toothpastes on inhibition of primary carious lesions. It is not clear weather simultaneous application of fluoride-releasing sealants and fluoridated toothpastes will improve the inhibition of caries or not. This study was performed to comparatively study the inhibitory effects of conventional and fluoride-releasing fissure sealants with and without exposure to fluoride toothpastes on initial carious lesions.

## Methods

In this in vitro study, 24 permanent first and second premolar teeth which had been extracted for orthodontic reasons were collected. The teeth were cleaned by fluoride-free prophylaxis paste and examined under a magnifier at ×4 (Helix hand-held magnifier) to make sure of the absence of cracks, caries, white or brown lesions, hypoplasia, fluorosis and pigmentation. Then, they were immersed in chloramine solution (Kimiapars, Iran) for 24 hours for disinfection. After disinfection, the teeth were kept in normal saline at room temperature (23‒27̊ºC) until the start of the study.

### Sample Preparation

The samples were randomly divided into three equal groups of A, B and C each (n=8). Using a fissurotomy bur (Teeskavan, Iran) and a high-speed handpiece (NSK, Japan), cavities with dimensions of 4*2*1.5 mm were prepared in the middle third of the buccal surface of each sample, so that the occlusogingival, mesiodistal and buccopalatal dimensions of the cavity were 2, 4 and 1.5 mm, respectively. In the cavities of the first group (group A), a fluoride-releasing fissure sealant (Embrac Wetbond Sealant, Pulpdent, USA) and in the second group (group B), a conventional fissure sealant (Master-Dent, Light-cured, Opaque, USA) were placed, and the third group (group C) did not undergo any intervention.

### Sealant Application

**Group A:** The cavities were etched with 37% phosphoric acid gel (Morva Etch, Iran) for 15 seconds, rinsed for 10 seconds and lightly dried with compressed air. The cavities were left slightly moist. Then the sealant was applied to the cavities and light-cured for 40 seconds.

**Group B:** The cavities were etched using 37% phosphoric acid gel for 30 seconds, rinsed for 10 seconds and dried with compressed air. The bonding agent (Single Bond, 3M ESPE, USA) was applied with a microbrush and light-cured for 20 seconds. Then the sealant material was placed in each cavity and light-cured for 40 seconds using a light-curing device (Dentamerica, Litex 680 A, Taiwan).

**Figure 1:**
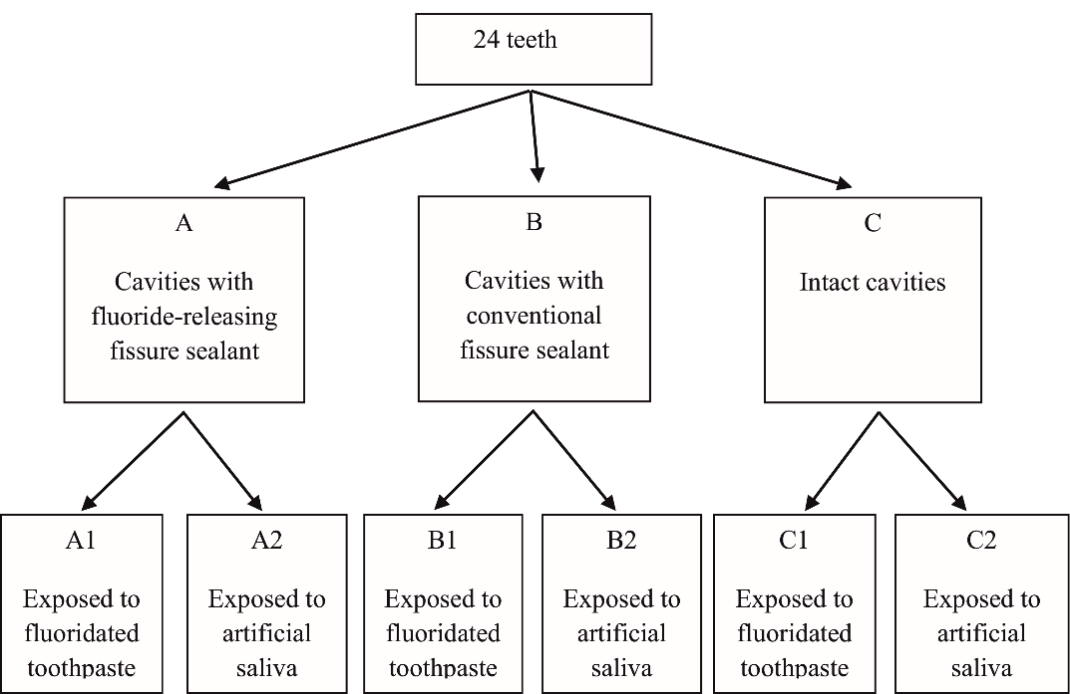
Main study groups (A, B and C) and subgroups (A1, A2, B1, B2, C1 and C2).

All the tooth surfaces were covered with acid-resistant varnish except a 1-mm rim around the cavities. The samples were placed in demineralizing solution‏ (2.2 µm of CaCl2, 1 m‏ of KOH, 2.2 µm of NaH2PO4, pH=4.5) for 96 hours to produce early lesions around the cavities. Then the samples were mounted in acrylic blocks (Acropars, Iran) and longitudinally cut in buccolingual direction using a microtome (Accutom-50; Struers, Copenhagen, Denmark) so that 100‒150-µm sections were achieved. After excluding defective sections, each main study group contained 8 sections. The depth of the created lesion in each sample was measured under a polarized light microscope at two points. All the surfaces of all the samples, except the surface of lesions, were covered with acid-resistant varnish. The samples in each of the three main groups were divided into two subgroups: group A (A1: fluoride-containing toothpaste, A2: artificial saliva (KIN-hydrate)), group B (B1: fluoride-containing toothpaste, B2: artificial saliva), group C (C1: fluoride-containing toothpaste, C2: artificial saliva) (Figure 1).

### pH Cycling

All the samples were placed in a pH-cycler for 10 days. Each sample was immersed in demineralizing solution (10 mL for each sample) twice a day for 3 hours, and in remineralizing solution (1.5 µm of CaCl_2_, 0.15 µm‏ of KCL, 0.9 µm of NaH_2_PO_4_, pH=7) (10 mL for each sample) for 2 hours between the two stages of demineralization. Each sample was placed in fluoride-containing toothpaste or artificial saliva (depending on its subgroup) for 60 seconds before the start of the first demineralization cycle and before and after the second demineralization cycle. Toothpaste solution was produced by mixing the toothpaste and water with a ratio of 1:3. Then, all the samples were immersed in remineralizing solution in an incubator at 37ºC overnight. After each phase of pH cycling, the samples were rinsed with deionized water for 30 seconds. The prepared solutions were replaced with fresh ones each day. After completion of pH cycling, the acid-resistant varnish was completely cleaned using acetone (Farmasi). Images of all the dental sections, before and after treatment, were obtained under a polarized light microscope (Heerbrugg, Switzerland), and the depths of the lesions in two similar points of each sample were measured.

### Statistical Analysis

The results of the study were reported using descriptive statistics (means ± standard deviations). In order to compare the depth of lesions by the three types of fissure sealants between the two types of exposures (toothpaste and artificial saliva), independent t-test or its non-parametric equivalent, Mann-Whitney U test was used. In order to compare the depth of lesions between the three types of fissure sealants using the two types of exposures (toothpaste and artificial saliva), two-way ANOVA was used in the case of normality of the data and homogeneity of the variances. In the case of significance of the results, an appropriate post hoc test was used for pair-wise comparisons between the groups. Significance of the study was considered at P˂0.05 and statistical analyses were carried out by SPSS 17.

## Results

The results showed that primary caries had the lowest depth in the fluoride-releasing fissure sealant group (603.12±51.73). The conventional fissure sealant group exhibited a mean decay depth of 889.37±56.38; primary caries had the highest depth in the samples without fissure sealant (1438.75±138.12) (Table 1). The difference between these three main study groups was significant (P˂0.001).

**Table 1:** Descriptive statistics of caries in different types of fissure sealants

	**Type of treatment**	**Number**	**Mean ± SD**	**P-value**
**Primary caries**	Fluoride-releasing sealant	16	603.12±51.73	<0.001
	Conventional sealant	16	889.37±56.38	
	Without fissure sealant	16	1438.75±138.12	
**Final caries**	Fluoride-releasing sealant	16	30±32.24	<0.001
	Conventional sealant	16	419.37±258.84	
	Without fissure seal	16	647.18±175.08	

Final caries exhibited the lowest depth in the fluoride-releasing fissure sealant group (30±32.24). The average depths of carious lesions were 419.37±258.84 and 647.18±175.08 in the conventional fissure sealant group and in the group without sealant, respectively (Table 1). The results a significant difference between these values (P˂0.001).

Pairwise comparisons of primary and secondary caries are presented in Table 2, indicating statistically significant differences between all the groups (P˂0.05).

**Table 2:** Pairwise comparisons of caries in different types of fissure sealants

	**Type of treatment**	**Mean difference**	** P-value**
**Primary caries**	Fluoride-releasing/Conventional	-286.25	<0.001
	Fluoride-releasing/Without sealant	-835.62	<0.001
	Conventional/Without sealant	-549.37	<0.001
**Final caries**	Fluoride-releasing/Conventional	-389.37	<0.001
	Fluoride-releasing/Without sealant	-617.18	<0.001
	Conventional/Without sealant	-227.81	0.019

Mann-Whitney U test was used to separately compare the depth of final caries after exposure to toothpaste and artificial saliva in each group. The results are presented in Table 3. Based on the results, the mean degree of caries in all the three groups was lower in toothpaste-exposed subgroups compared to the saliva-exposed subgroups. The difference of caries depth between the two types of exposures in the conventional fissure sealant group and the group without fissure sealant was significant (P˂0.001). However, the difference was not significant in the fluoride-releasing fissure sealant group (P=0.721).

**Table 3:** Pair-wise comparisons of caries depths in different types of fissure sealants

	**Type of exposure**	**Mean ± SD**	**P-value**
**Fluoride-releasing sealant**	Toothpaste	26.25±35.63	0.721
	Artificial saliva	33.75±35.43	
**Conventional sealant**	Toothpaste	196.25±165.86	<0.001
	Artificial saliva	642.5±47.65	
**Without fissure sealant**	Toothpaste	485.62±41.95	<0.001
	Artificial saliva	808.75±62.28	

## Discussion

Remineralization of initial caries without using aggressive “cut & fill” techniques is a major goal in modern dentistry. Remineralization process can occur in the presence of materials which release fluoride, calcium and phosphorus ions. Pit and fissure sealants are highly effective means of preventing carious lesions in deep pits and fissures. Incorporation of fluoride in sealants has been a controversial issue.

Efficacy of fluoride-releasing sealants has been demonstrated in a number of investigations. A study by Jensen ME showed that a fluoride-releasing fissure sealant significantly reduces the degree of demineralization of tooth enamel in the presence of materials.^4^ Another study by Hicks MJ et al^15^ showed that fluoride-releasing fissure sealants exhibit decay-inhibition effects by significantly reducing the depth of lesions on the enamel surface adjacent to the fluoride-releasing sealant. Also, a study by Locker et al^16^ showed that use of fluoride-containing fissure sealants is effective in restoring primary tooth and preventing primary decay.

Comparison of the inhibitory effect of fluoride-releasing and conventional sealants on primary caries in some studies has shown no significant differences between them;^11,17,18^ however, some have demonstrated higher efficacy of fluoride-releasing sealants.^14,19^ Some studies have shown that the inhibitory effect of fluoride-releasing sealants on adjacent primary caries is limited to the glass-ionomer-based sealants, and resin-based ones cannot yield significant results,^11,17,18^ indicating that fluoride release is difficult from resin-based sealants due to their lower hydrophilicity.^18,20,21^ In the current study, we compared the inhibitory effects of a resin-based fluoride-releasing sealant with a conventional sealant and according to the results, both primary and final caries depths in the fluoride-releasing fissure sealant group were significantly less than those in other groups. Therefore, it seems that fluoride-releasing sealants are more effective than conventional sealants in inhibiting caries.

The results of this study indicated that caries depth in the presence of different types of fissure sealants were significantly different in terms of the type of exposure, so that it was found that the average rate of decay in all the three groups in toothpaste subgroups was less than that in the artificial saliva group; in conventional samples and the samples without fissure sealant, these values were significant. This confirms the results of several investigations which have shown the remineralizing effect of fluoridated toothpastes on primary caries.^22,23^ However, in the fluoride-releasing fissure sealant group, the difference between the samples exposed to toothpaste and saliva was not significant.

## Conclusion

It can be concluded from the results of our study and other studies that incorporation of fluoride into fissure sealants can be effective in inhibiting dental caries and in general, it might lead to decreased prevalence of dental caries. However, fluoridated tooth paste use in conjunction with fluoride-releasing fissure sealant does not exhibit additional positive effects. Fluoridated tooth paste is effective in inhibiting primary caries only in teeth which have conventional sealants or in teeth without a sealant. It seems that fluoride released from fluoride-releasing sealants overwhelms the remineralizing capacity of fluoride released from toothpastes in the same tooth.

## Acknowledgments

We would like to express our thanks to Tabriz Faculty of Physics for their cooperation.

## Authors’ Contributions

ZKS: designing the study protocol, guidance in all stages of study performance, manuscript preparation, ES: performance of the study (caries depth measurements using polarized light microscope) MA: performing the study, manuscript preparation, MMS: designing the study protocol, SHHN: manuscript preparation.

## Funding

Not applicable.

## Conflict of Interests

The authors declare no competing interests with regards to the authorship and/or publication of this article.

## Ethics Approval

Tabriz Medical Sciences University Ethics Committee approved the study protocol.
